# DNAJB4/HLJ1 deficiency sensitizes diethylnitrosamine-induced hepatocarcinogenesis with peritumoral STAT3 activation

**DOI:** 10.1007/s10565-024-09978-y

**Published:** 2024-12-30

**Authors:** Wei-Jia Luo, Wei-Lun Hsu, Chih-Yun Lu, Min-Hui Chien, Jung-Hsuan Chang, Kang-Yi Su

**Affiliations:** 1 Department of Clinical Laboratory Sciences and Medical Biotechnology, College of Medicine, National Taiwan University, Taipei, Taiwan; 2 Genome and Systems Biology Degree Program, National Taiwan University and Academia Sinica, Taipei, Taiwan

**Keywords:** Hepatocellular carcinoma, DNAJB4, HLJ1, Diethylnitrosamine, DEN, STAT3

## Abstract

Environmental chemicals and toxins are known to impact human health and contribute to cancer developments. Among these, genotoxins induce genetic mutations critical for cancer initiation. In the liver, proliferation serves not only as a compensatory mechanism for tissue repair but also as a potential risk factor for the progression of premalignant lesions. The role of Human Liver DnaJ-Like Protein (DNAJB4/HLJ1), a stress-responsive heat shock protein 40, in genotoxin-induced liver carcinogenesis remains unexplored. Using whole-genome transcriptomic analysis, we demonstrate that HLJ1 deficiency in mice results in altered gene signatures enriched in pathways associated with chemically induced liver cancer and IL-6/STAT3 signaling activation. Employing diethylnitrosamine (DEN) as a carcinogen, we further reveal that STAT3 and H2AX phosphorylation induced by short-term DEN treatment are amplified in HLJ1-deficient mice. In long-term DEN experiments, HLJ1 deletion enhances tumor proliferation and progression, accompanied by pronounced STAT3 phosphorylation in normal tissues rather than in tumor regions. The tumor-suppressive role of peritumoral HLJ1 is validated through the transplantation of HLJ1-wildtype B16F1 and LLC cancer cell lines into syngeneic HLJ1-deficient mice, which exhibits an augmented tumorigenic phenotype compared to wildtype controls. This study uncovers a previously unrecognized role of HLJ1 in suppressing liver carcinogenesis via the downregulation of STAT3 signaling in peritumoral normal cells. These findings suggest that HLJ1 reinforcement represents a promising strategy for liver cancer treatment and prevention.

**Graphical Abstract:**

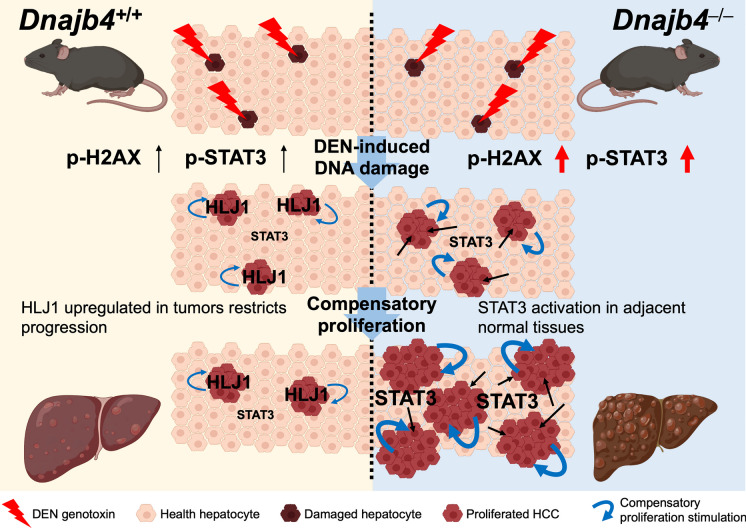

**Supplementary information:**

The online version contains supplementary material available at 10.1007/s10565-024-09978-y.

## Introduction

Accumulating evidence has substantially advanced our understanding of how exposure to environmental toxins and chemicals negatively impacts human health and contributes to cancer incidence and progression. Genotoxicity, the ability of certain substances to damage cellular genetic material, compromises cellular integrity and leads to mutations that increase cancer risk (Ren et al. 2017). The WHO estimates that approximately 20% of cancer cases are attributable to environmental factors, including chemicals and toxins (Collaborators 2018). The liver, as the primary organ for detoxifying and processing endogenous and exogenous toxins and metabolic byproducts, plays a central role in responding to environmental challenges (Liska 1998). Notably, the liver possesses a remarkable regenerative capacity, enabling it to replace lost cells through compensatory proliferation following partial tissue loss or cellular damage (Hora and Wuestefeld 2023). However, this regenerative process can also promote the progression of focal or clonal premalignant lesions into malignant stages. Understanding the cellular responses and regulatory networks within the liver microenvironment following exposure to chemicals or toxins is essential for advancing cancer prevention and treatment strategies.

Heat shock proteins (HSPs) function as molecular chaperones to help cells manage environmental stresses, including exposure to carcinogens. Human Liver DnaJ-Like Protein (HLJ1), encoded by the *Dnajb4* gene in mice, is a heat shock protein upregulated in response to various stressors, such as heat shock, endotoxins, and high-fat media (Hoe et al. 1998; Luo et al. 2022; Luo et al. 2018). HLJ1 is widely recognized as a tumor suppressor, as its upregulation suppresses cancer cell invasion (Liu et al. 2014; Tsai et al. 2006; Wang et al. 2005). Recent studies have highlighted its biological functions in the liver, including its role in immune modulation by regulating IL-12 maturation in liver macrophages (Luo et al. 2022). Single-cell RNA sequencing data further reveal HLJ1’s involvement in IFN-γ-activated signaling pathways, MHC class-I–related signals, and MIF signaling pathways in macrophages and dendritic cells (Luo et al. 2022). Despite these insights, HLJ1’s role in chemicals or genotoxins-induced liver carcinogenesis, particularly during early initiation, remains unclear. Tumor initiation caused by carcinogenic toxins involves multifactorial processes that are time- and dose-dependent. The nature of genetic mutations, whether drivers or non-drivers, is also a critical consideration. Chronic carcinogen exposure can increase the accumulation of mutations over time, potentially leading to aggressive malignant tumors. The response to carcinogen-induced stress, conserved across mammals, is essential for preventing cancer initiation and progression (Zhang et al. 2024). However, the role of endogenous stress proteins in carcinogen-induced tumor initiation and progression remains relatively underexplored. Investigating whether HLJ1 modulates stress responses induced by carcinogenic toxins and affects tumor progression is thus a worthwhile area of research.

These studies suggest that HLJ1 plays a critical role in maintaining liver function homeostasis, potentially impacting liver cancer development. In this study, we used HLJ1-knockout (*Dnajb4*^–/–^) mice and demonstrated that HLJ1 deficiency leads to hepatic gene signatures linked to chemical-induced liver cancer and IL-6/STAT3 signaling. Furthermore, the absence of HLJ1 contributes to DEN-induced carcinogenesis and proliferation, likely due to increased phosphorylation of STAT3 in adjacent normal tissues rather than in tumor tissues. This study uncovers the previously unknown role of HLJ1 in suppressing liver cancer with peritumoral STAT3 inhibition, and thus HLJ1 reinforcement could be a promising strategy for both liver cancer treatment and prevention.

## Results

### HLJ1 potentially participates in chemical-induced carcinogenesis through IL-6/STAT3 signaling

**Fig. 1:**
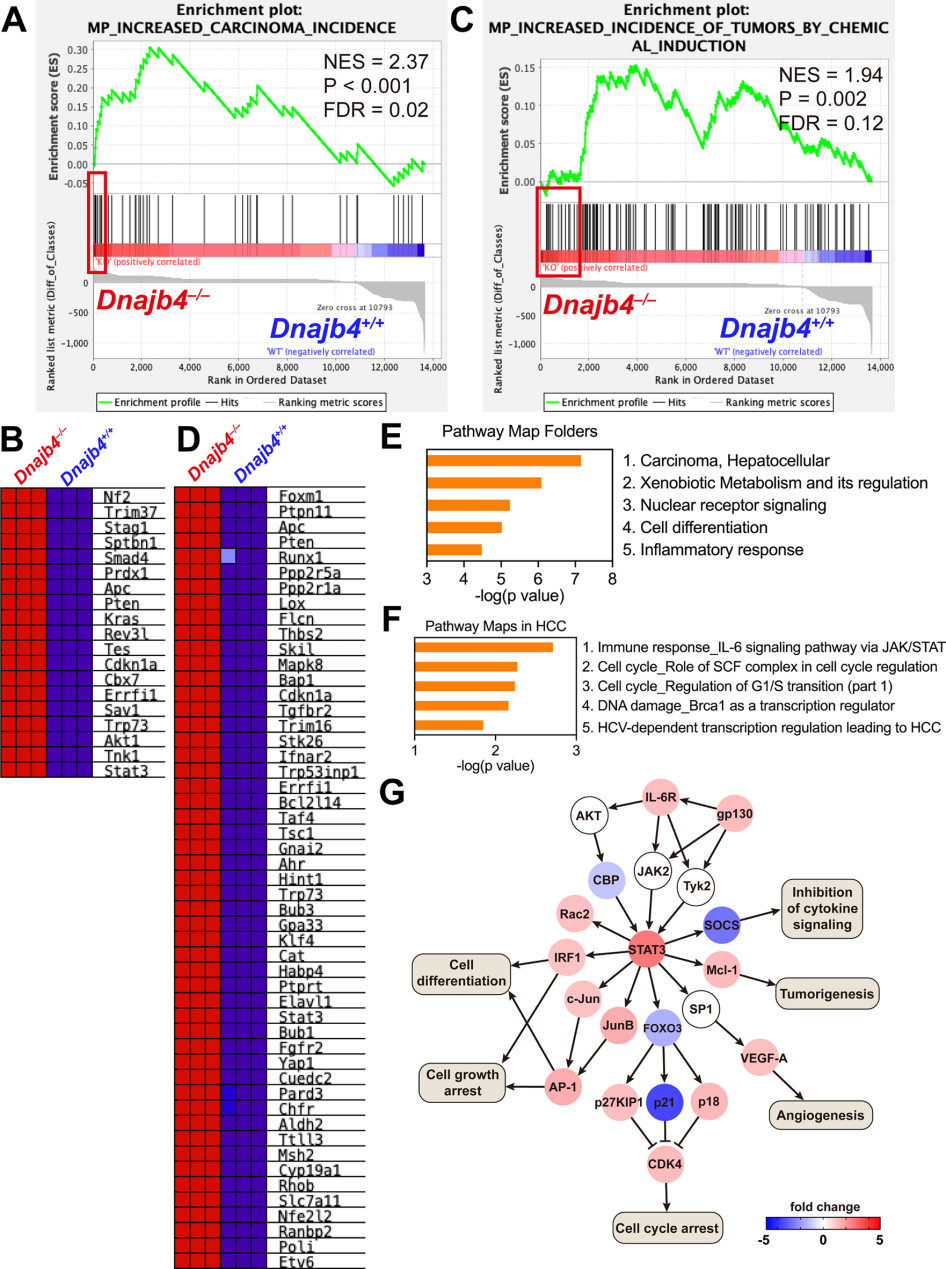
cDNA microarray analysis of HLJ1-related biological pathways in liver cancer. mRNA was extracted from the liver of *Dnajb4*^+/+^ and *Dnajb4*^–/–^ mice aged 6–8 weeks (n = 3 per group) for cDNA microarray and GSEA analysis. GSEA identified significantly enriched gene sets, including (**A**) “increased carcinoma incidence” and (**C**) “increased incidence of tumors by chemical induction,” along with their corresponding core enrichment genes (**B** and **D**, respectively). **E** The top five ranked categories from “Pathway map folders” based on gene enrichment analysis of 267 selected genes. **F** The top five enriched pathway maps within the folder “hepatocellular carcinoma”, ranked by *P*-values. **G** Network construction analysis of differentially expressed genes enriched in the IL-6 signaling pathway via JAK/STAT. Red boxes indicate genes identified as positive core enrichments in *Dnajb4*^**–/–**^ mice

We investigated whether HLJ1, a stress-responsive heat shock protein, potentially participated in maintaining liver function homeostasis, particularly in carcinogenesis. Whole-genome transcriptome assays were conducted, followed by enrichment analysis and regulatory network construction, using liver mRNA extracted from HLJ1-wildtype (*Dnajb4*^+/+^) and HLJ1-knockout (*Dnajb4*^–/–^) mice (Fig. 1). Gene set enrichment analysis (GSEA) focusing on tumor phenotype ontology, encompassing 92 gene sets, revealed significantly enrichment in two gene sets between two genotypes (Fig. S1). The first one regarding increased carcinoma incidence was significantly positively enriched in *Dnajb4*^–/–^ mice (Fig. 1A), with 19 of 40 core-enriched genes (the red box in Fig. 1A) identified (Fig. 1B and Table S1). The second gene set indicated upregulated genes in *Dnajb4*^–/–^ mice linked to pathways associated with increased incidence of tumors by chemical induction (Fig. 1C), with 51 of 116 core-enriched genes (the red box in Fig. 1C) identified (Fig. 1D and Table S2). These findings suggest that mice deficient in HLJ1 may be susceptible to environmental stress-induced carcinogenesis, particularly from chemicals. To validate this hypothesis, we investigated the underlying molecular pathways regulated by HLJ1. A total of 267 differentially expressed genes (up-/down-regulated by at least twofold) between the genotypes were selected for subsequent pathway analysis and network construction (Fig. 1E). Enrichment analysis of “pathway map folder” revealed that these genes were significantly enriched in pathways associated with “hepatocellular carcinoma”, indicating that HLJ1 deficiency may activate HCC-related genes. Notably, pathways involved in xenobiotic metabolism and regulation were significantly upregulated in the absence of HLJ1, suggesting that *Dnajb4*^–/–^ mice may exhibit heightened sensitivity to chemical-induced hepatocarcinogenesis. Further investigation of the HCC category highlighted enriched pathway maps, including immune responses mediated by IL-6/JAK/STAT signaling, cell cycle regulation of SCF complex and G1/S transition, and DNA damage response pathways (Fig. 1F). To identify potential regulatory networks influenced by HLJ1 and contributing to HCC, we used the differentially expressed genes to construct a network based on the canonical IL-6/JAK/STAT pathway, which ranked highest in significance within HCC pathway map folders (Fig. 1G). This network identified STAT3 as a core transcriptional factor, upregulated after HLJ1 deletion, regulating several downstream targets, including SOCS, Mcl-1, FOXO3, JunB, c-Jun, IRF1, and Rac2. These genes contribute to various liver cancer phenotypes, including cell growth and cell cycle arrest, cell differentiation, inhibition of cytokine signaling, tumorigenesis, and even angiogenesis. These results suggest that HLJ1 deficiency enhances chemical-induced liver carcinogenesis by altering gene signatures and activating pathways, particularly those involving IL-6/STAT3 signaling.

### DEN-induced phosphorylation of STAT3 and H2AX is enhanced in *Dnajb4*^–/–^ mice 

**Fig. 2:**
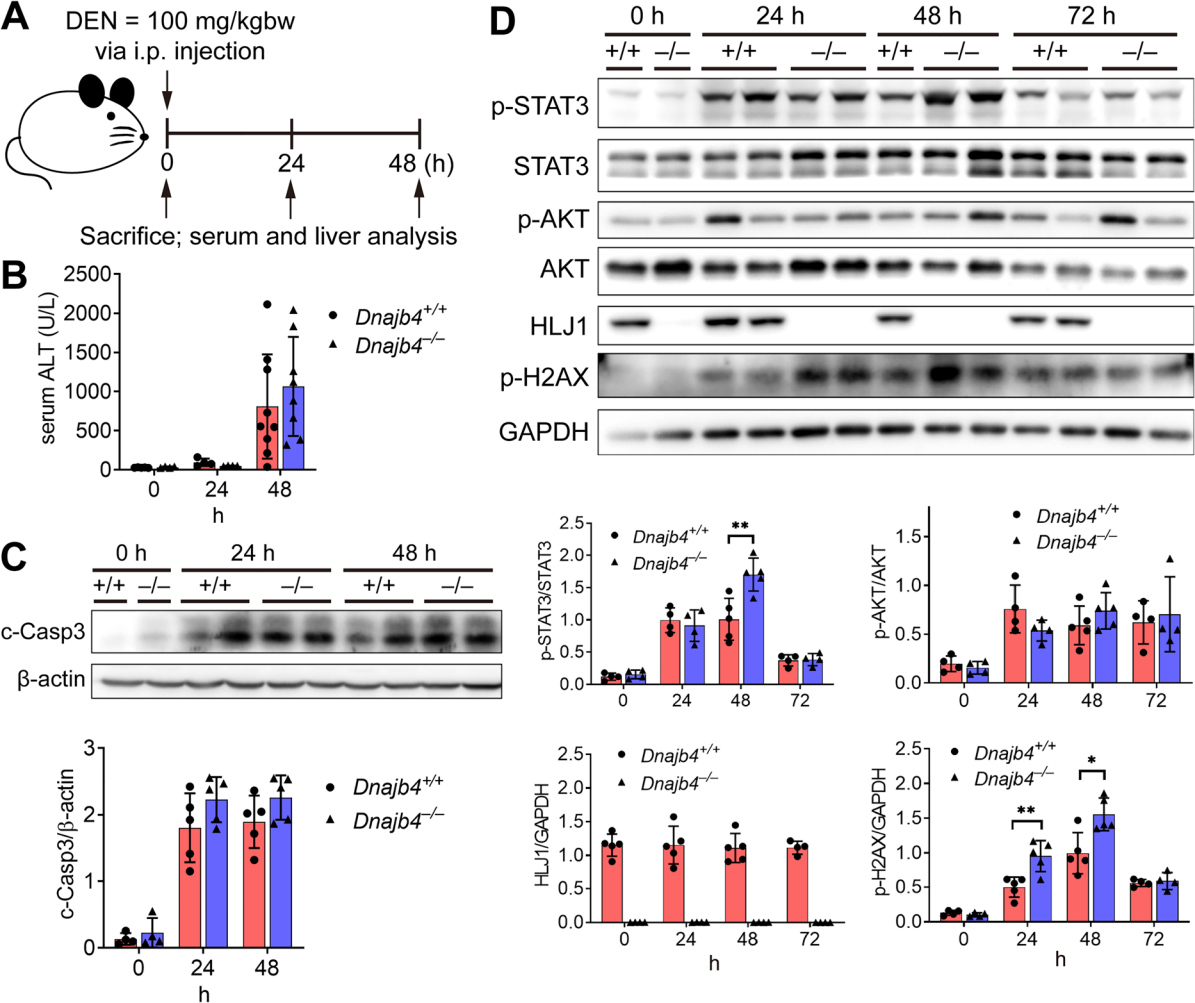
HLJ1 deletion enhances p-STAT3 and H2AX under short-term DEN stress. **A** *Dnajb4*^+/+^ and *Dnajb4*^–/–^ mice were administrated a single dose of DEN (100 mg/kg) at 6–8 weeks of age and sacrificed at the indicated time points for proteins extraction from whole liver lysates. **B** Serum ALT levels in *Dnajb4*^+/+^ and *Dnajb4*^–/–^ mice at 0, 24, and 48 h post-DEN (100 mg/kg) administration. **C** Expression levels of cleaved caspase-3 in DEN-treated liver of *Dnajb4*^+/+^ (+ / +) and *Dnajb4*^–/–^ (–/–) mice were assessed by western blotting. **D** Protein levels of HLJ1 and phosphorylated STAT3, AKT, and H2AX in *Dnajb4*^+/+^ (+ / +) and *Dnajb4*^–/–^ (–/–) mice livers. Representative 1 ~ 2 samples out of n = 4 per group are shown. β-actin and GAPDH serve as loading controls. Data are presented as means ± SD. * *P* ≤ 0.05; ** *P* ≤ 0.01

Given that HLJ1 deficiency potentially alters hepatic gene expression patterns related to xenobiotic metabolism and chemical-induced tumor incidence, we utilized diethylnitrosamine (DEN), a well-established carcinogen that induces mouse liver cancer resembling human HCC (Lee et al. 2004), to clarify the role of HLJ1 in hepatocarcinogenesis. The short-term effects of DEN were assessed by administrating a 100 mg/kg dosage and sacrificing mice 24 or 48 h post-injection (Fig. 2A). To evaluate liver damage, we analyzed markers of hepatic injury, as DEN metabolism generates reactive oxygen species (ROS) via the cytochrome P450 system, causing hepatocyte death and triggering compensatory proliferation (Kang et al. 2007). Serum transaminase levels increased in response to DEN but showed no significant difference between genotypes (Fig. 2B). Additionally, the expression level of Cyp2E1, an enzyme primarily responsible for hepatic DEN metabolism, was unaffected by HLJ1 deletion (Fig. S2A). Although DEN induces ROS-mediated liver damage, the expression level of Hmox-1, an enzyme responsive to oxidative stress, also remained unchanged after HLJ1 deletion (Fig. S2B). Analysis of long-term oxidative stress markers GSTP1, GCLC, GCLM, and NQO1 in normal parts adjacent to HCC using the TCGA public database revealed little correlation with the expression of HLJ1 (Fig. S3).

To examine apoptosis following DEN treatment, we evaluated cleaved caspase-3 levels (Fig. 2C), Although *Dnajb4*^–/–^ mice exhibited higher levels of cleaved caspase-3 than *Dnajb4*^+/+^ mice, the difference was not statistically significant. Similarly, the transcriptional levels of Bax were unaffected by HLJ1 deletion (Fig. S2C), indicating that HLJ1 does not impact the intrinsic apoptotic pathway. Collectively, these findings suggest that HLJ1 does not influence DEN-induced liver damage during short-term challenges. Since STAT3 phosphorylation (p-STAT3) can occur in response to cytokine IL-6 secreted by macrophages during DEN-induced tumor initiation and thus reflect liver inflammation, we investigated whether p-STAT3 levels were altered in *Dnajb4*^–/–^ mice post-DEN injection (Fig. 2D). The results showed significantly elevated p-STAT3 levels in *Dnajb4*^–/–^ mice compared to *Dnajb4*^+/+^ mice at 48 h post-injection. However, IL-6 expression levels remained unchanged in the absence of HLJ1 (Fig. S2D), suggesting an IL-6-independent pathway mediating STAT3 activation. As DEN metabolism generates electrophilic products causing DNA damage by reacting with nucleophilic DNA bases, we next analyzed p-H2AX, a marker of DNA damage, in mice post-DEN treatment. Compared to *Dnajb4*^+/+^ controls, *Dnajb4*^–/–^ mice displayed significantly higher levels of p-H2AX 24 and 48 h after DEN challenge (Fig. 2D). By 72 h, p-H2AX expression decreased, indicating the completion of damage repair. These findings suggest that HLJ1 attenuates short-term DEN-induced STAT3 phosphorylation and DNA damage responses. HLJ1 may play a modulatory role in stress responses triggered by chemical carcinogens.

### Tumor burden is enhanced in HLJ1-deficient mice following long-term DEN treatment

**Fig. 3:**
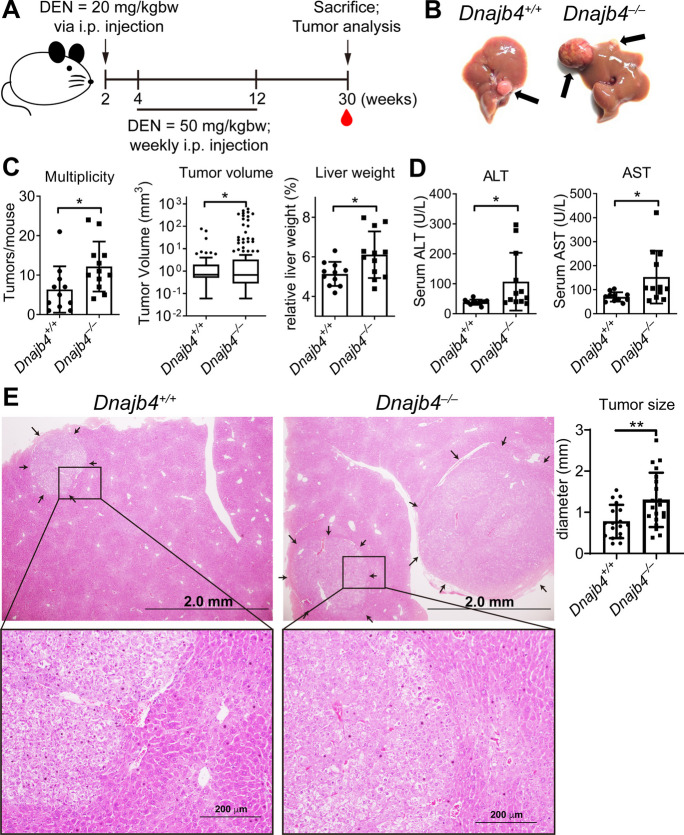
HLJ1 deletion increases tumor volume and multiplicity. **A** *Dnajb4*^+/+^ and *Dnajb4*^–/–^ mice received a single 20 mg/kg DEN injection at 2 weeks of age, followed by weekly 50 mg/kg DEN administration from 4 to 12 weeks of age. Mice were sacrificed at 30 weeks for tumor assessment. **B** Representative images of livers from male *Dnajb4*^+/+^ and *Dnajb4*^–/–^ mice. Arrows indicate macroscopic tumors. **C** Quantification of tumor multiplicity (tumors per mouse), tumor volume (1/2 × length × width^2^), and relative liver weight (liver/body weight). Tumor volume is displayed as Whiskers Tukey box plots. Data represent mean ± SD from 11 ~ 12 mice per group. **D** Serum ALT and AST levels measured in 11 ~ 12 mice per group. **E** Microscopic lesions analyzed from H&E-stained liver sections. Scale bar = 2 mm. Data represents mean ± SD obtained from at least three tumors per mouse and n = 11 ~ 12 mice per group. Tumor size and volume were compared using Student’s t test. * *P* ≤ 0.05; ** *P* ≤ 0.01

Based on the findings from short-term experiment, we further investigated whether HLJ1 significantly influences DEN-induced liver cancer formation and progression. Long-term DEN administration in mice was followed by histopathological analysis. Two-week-old mice of both genotypes received a single dose of DEN (20 mg/kg, i.p.), followed by weekly high-dose DEN (50 mg/kg, i.p.) starting immediately after weaning until 12 weeks of age. Mice were sacrificed for tumor evaluation at 30 weeks (Fig. 3A). The results showed *Dnajb4*^–/–^ mice exhibited significantly higher macroscopic tumor burdens compared to *Dnajb4*^+/+^ mice (Fig. 3B). Tumor multiplicity, volume, and relative liver weight were also significantly elevated in *Dnajb4*^–/–^ mice (Fig. 3C). Serum levels of ALT and AST were notably higher in *Dnajb4*^–/–^ than *Dnajb4*^+/+^ mice (Fig. 3D), indicating augmented liver damage. The differences in ALT/AST were not observed during the short-term DEN challenge (Fig. 2), as the long-term constitutive liver damage resulted from tumor growth reflects severity. To further assess the impact of HLJ1 on tumor progression, we quantified the size of microscopic tumor foci in liver sections (Fig. 3E). *Dnajb4*^–/–^ mice exhibited significantly larger microscopic tumors than *Dnajb4*^+/+^ mice. To confirm these findings, we used an additional experimental model involving phenobarbital (PB), a common tumor promotor used post-DEN induction. Mice received a single DEN dose at 2 weeks of age, followed by 500 ppm PB in drinking water starting at 4 weeks until 55 weeks of age (Fig. S4A). Consistent with the primary model, *Dnajb4*^–/–^ mice exhibited a 100% incidence rate, greater macroscopic tumor burden, and higher multiplicity (number of tumors) than *Dnajb4*^+/+^ mice (Figs. S4B and C). HLJ1 deletion also resulted in slightly elevated tumor size, serum ALT and AST levels, and relative liver weight in the DEN/PB model (Fig. S4C). These results collectively demonstrate that tumor burden and progression are significantly exacerbated in *Dnajb4*^–/–^ compared to *Dnajb4*^+/+^ mice. Across multiple analytical metrics and experimental models, the absence of HLJ1 contributes to enhanced carcinogen-induced tumor progression, underscoring the critical role of HLJ1 in mitigating liver tumor development.

### HLJ1 deficiency leads to tumor proliferation with enhanced p-STAT3 signaling in normal liver adjacent to tumor tissues

**Fig. 4:**
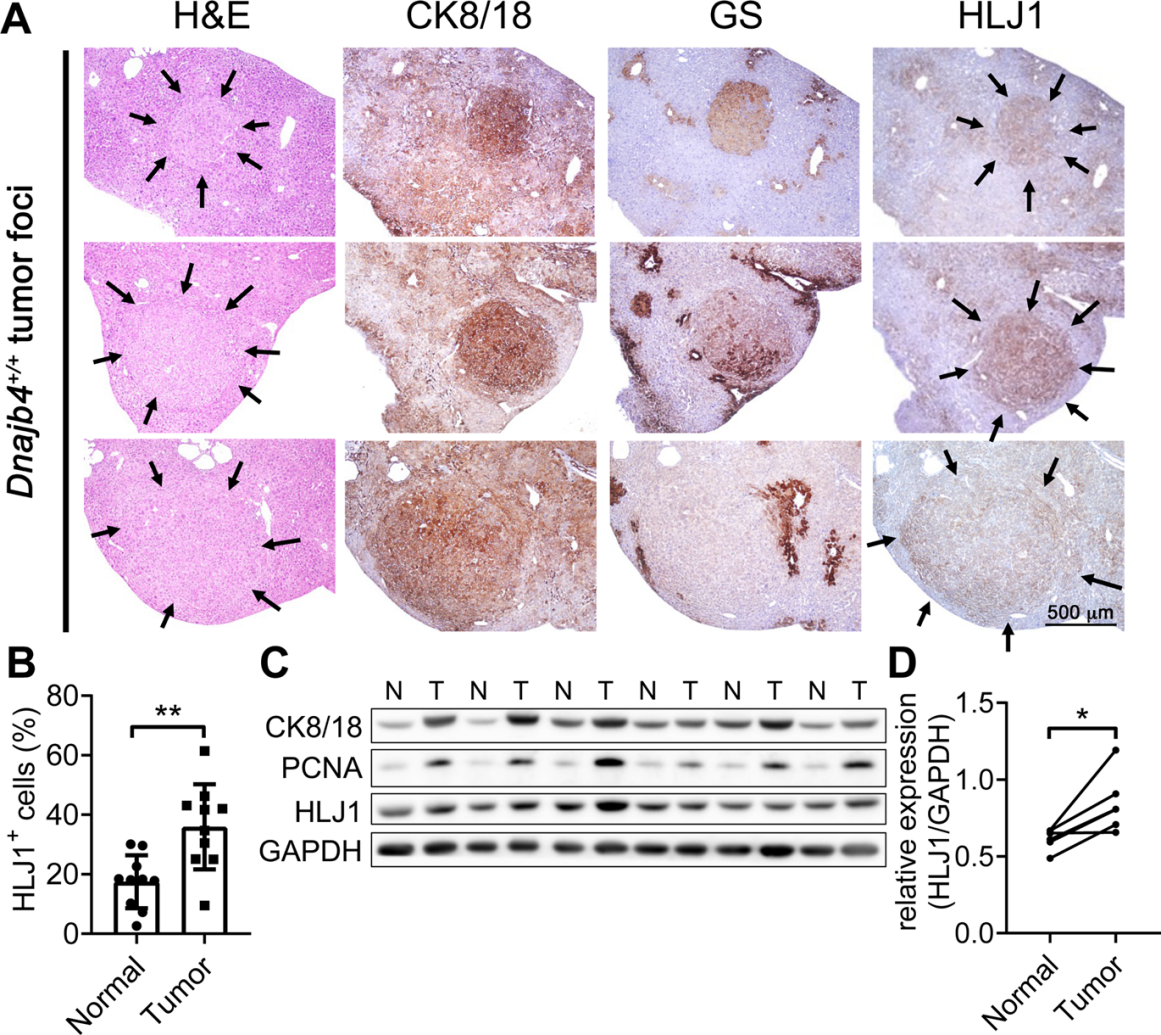
HLJ1 expression is elevated in tumors compared to adjacent normal tissues. **A** HLJ1 expression levels were assessed in the tumor and adjacent normal liver tissues from *Dnajb4*^+/+^ mice. Serial liver sections were strained with H&E and IHC for HLJ1. Cytokeratin 8/18 (CK8/18) and glutamine synthetase (GS) served as tumor markers. Arrows indicate individual microscopic tumors. Scale bar = 500 μm. **B** Quantification of HLJ1-positive cells in tumor and adjacent normal tissues. **C** Protein levels of HLJ1 were analyzed from excised macroscopic tumors and paired adjacent normal tissues. CK8/18 and PCNA were used as tumor markers, while GAPDH served as loading control. **D** Band intensities of HLJ1 were quantified, and significance was evaluated using a paired Student’s t-test. * *P* ≤ 0.05; ** *P* ≤ 0.01

To clarify the role of HLJ1 in the liver microenvironment and characterize its spatial expression during tumor progression, we performed immunohistochemical staining on serial liver sections from *Dnajb4*^+/+^ mice (Fig. 4A). CK8/18 and GS were used as markers for proliferation in basophilic foci and tumors and for Wnt/β-catenin signal transduction activation, respectively (Buechel et al. 2021; Kakehashi et al. 2010). Interestingly, HLJ1 expression was enriched in and colocalized with tumor foci compared to adjacent normal liver tissues (Fig. 4B). Quantification of HLJ1 protein levels from isolated macroscopic tumors and paired non-tumor liver tissues confirmed significantly higher HLJ1 expression in tumors than in normal liver tissues (Figs. 4C and D). In addition, PCNA, a marker of cell proliferation, was also highly expressed in tumor tissues compared to adjacent normal tissues. Analysis of publicly available TCGA data showed slightly higher HLJ1 expression in human liver tumors compared to normal tissues (Fig. S5A), while HLJ1 expression levels did not differ significantly across major HCC stages (Fig. S5B). Kaplan–Meier analysis revealed a slight association between higher HLJ1 expression in tumors and poorer overall survival, while no significant correlation was found with progression-free survival (Figs. S5C and D). These findings suggest heterogeneous HLJ1 expression within tumor and normal liver tissues, indicating distinct functional roles in these regions.

**Fig. 5:**
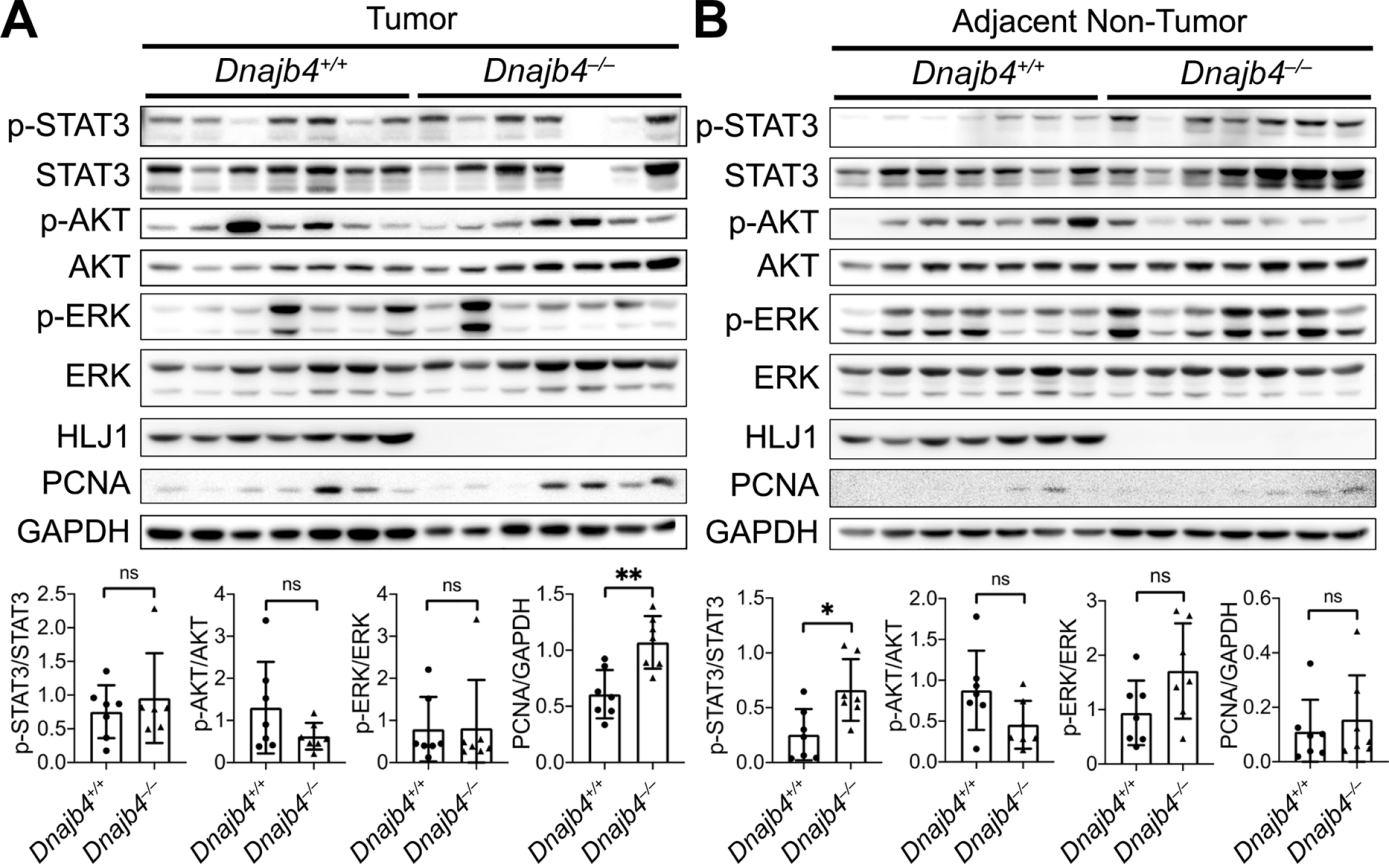
Elevated p-STAT3 levels in the adjacent non-tumor regions of *Dnajb4*^–/–^ mice. Western blot analysis of proteins extracted from paired (**A**) macroscopic tumor and (**B**) adjacent non-tumor tissues of 30-week-old DEN-treated *Dnajb4*^+/+^ and *Dnajb4*^–/–^ mice. Expression levels of HLJ1 and tumor growth-related proteins, including STAT3, AKT, ERK, and PCNA, were evaluated. GAPDH served as a loading control. Data represent the mean ± SD from n = 7 mice. * *P* ≤ 0.05; ** *P* ≤ 0.01; ns, not significant

Activated STAT3, AKT, and ERK are crucial for maintaining a pro-carcinogenic inflammatory microenvironment during not only initiation of malignant transformation but also cancer progression. We analyzed the expression and phosphorylation levels of these molecules in tumor and normal tissues (Fig. 5). In tumor tissues, the phosphorylation levels of STAT3, AKT, and ERK were similar between *Dnajb4*^+/+^ and *Dnajb4*^–/–^ mice, indicating universal activation of these pathways (Fig. 5A). However, tumor tissues in *Dnajb4*^–/–^ mice showed enhanced cell proliferative activity, as indicated by increased PCNA staining. In adjacent normal liver tissues, *Dnajb4*^–/–^ mice showed significantly upregulated p-STAT3 levels compared to *Dnajb4*^+/+^ mice (Fig. 5B). In contrast, the activation levels of AKT and ERK remained unchanged in normal tissues following HLJ1 deletion. Further analysis of the STAT3 signaling pathway in human normal liver tissues adjacent to tumors revealed a significant negative correlation between HLJ1 and SOCS3 expression (Fig. S6A). However, no correlation was observed between HLJ1 and upstream factors contributing to STAT3 activation, such as IL-6, LIF, ROS, and EGFR (Figs. S6B-E). These findings suggest that HLJ1 may directly impact and downregulate the STAT3/SOCS3 axis independently of upstream effectors.

To explore the potential broader implications of HLJ1 in liver cancer progression, we performed enrichment analysis using the top 1000 genes most similar to HLJ1 in the TCGA human liver cancer database. The gene ontology process revealed genes enriched in pathways associated with metabolism (Fig. S7A). Pathway maps and network analysis further indicated enrichment in DNA damage pathways, including intra S-phase checkpoint and double-strand break repair (Figs. S7B and C). These results demonstrate that HLJ1 deficiency in the normal tissue adjacent to tumors is associated with elevated p-STAT3 levels, potentially contributing to compensatory proliferation following DEN-induced damage. This supports the hypothesis that HLJ1 plays a suppressive role in liver carcinogenesis.

### HLJ1 deficiency in normal liver microenvironment creates a niche favorable for tumor growth

**Fig. 6:**
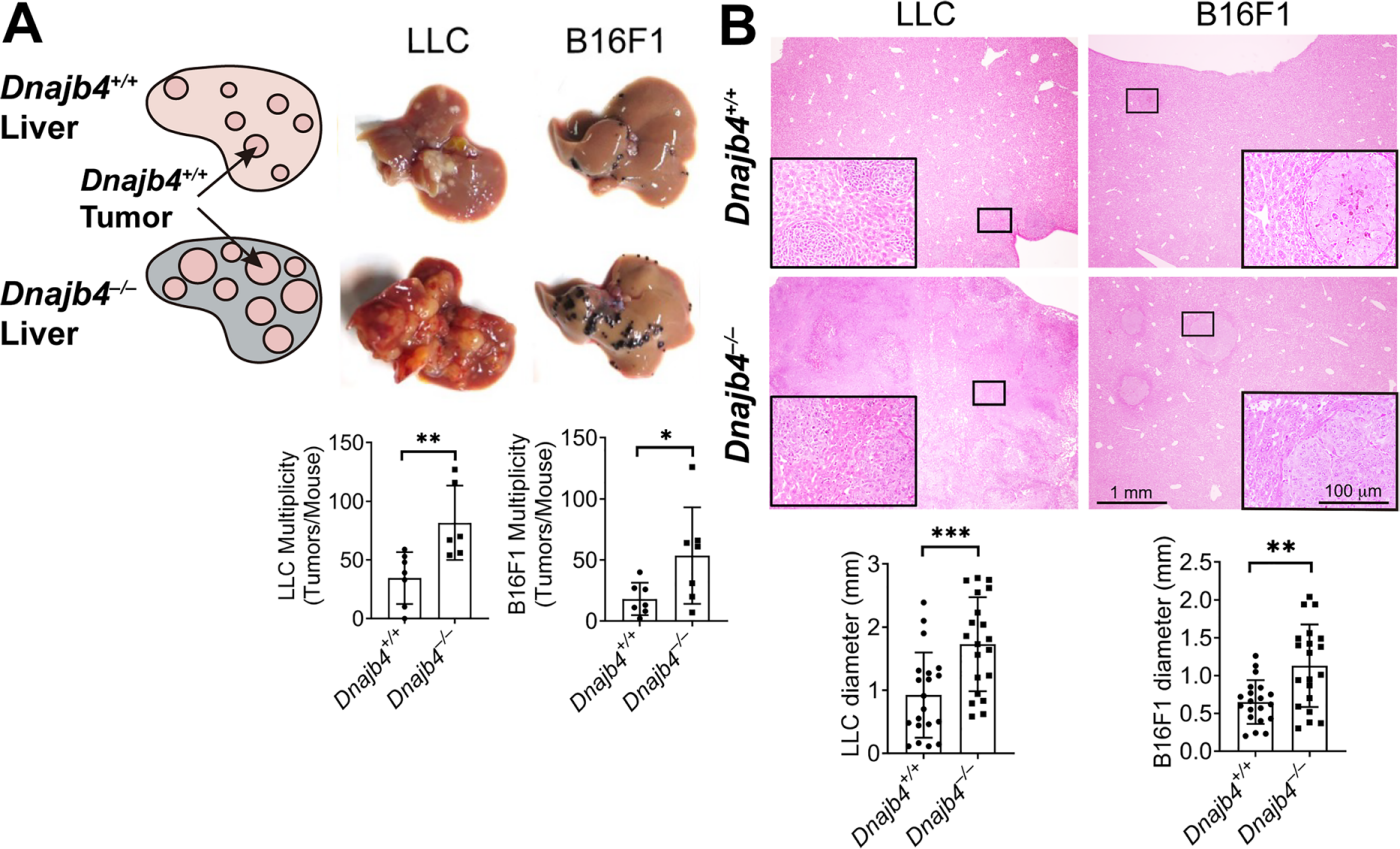
Enhanced growth of transplanted LLC and B16F1 cells in HLJ1-deficient livers. **A** *Dnajb4*^+/+^ and *Dnajb4*^–/–^ mice were intrasplenically injected with B16F1 and LLC cell lines expressing wild-type HLJ1. Macroscopic liver tumor lesions were evaluated 13 and 18 days post-transplantation. Data are presented as mean ± SD from n = 6–7 mice per group. **B** Microscopic tumor lesions were assessed from H&E-stained liver sections. Data represent mean ± SD obtained from n = 3 tumors per mouse and 6–7 mice per group. * *P* ≤ 0.05; ** *P* ≤ 0.01; *** *P* ≤ 0.001

Significantly higher p-STAT3 expression levels were observed in the normal liver tissues of *Dnajb4*^*–/–*^ mice compared to *Dnajb4*^+*/*+^ mice. This raised the question of whether an HLJ1-deficient environment alone could establish a growth-advantageous niche for tumor cells. To address this, syngeneic Lewis lung carcinoma (LLC) and melanoma F1 (B16F1) cells, both of which express normal levels of HLJ1, were implanted via intrasplenic injection. This method allowed tumor cells to migrate to and reside within the liver, enabling us to evaluate tumor growth under HLJ1-deficient conditions (Fig. 6A). The results demonstrated that *Dnajb4*^*–/–*^ mice developed significantly larger macroscopic tumors than *Dnajb4*^+*/*+^ mice (Fig. 6A, upper panel). Quantification showed that the multiplicity of macroscopic LLC and B16F1 tumors in *Dnajb4*^*–/–*^ mice was significantly higher than in *Dnajb4*^+/+^ mice (Fig. 6A, lower panel). Microscopically, the size of LLC and B16F1 tumor lesions in the liver was significantly larger in *Dnajb4*^*–/–*^ mice than in *Dnajb4*^+/+^ mice (Fig. 6B). These results suggested that HLJ1 in the normal part of the liver microenvironment suppressed tumor growth and carcinogenesis.

## Discussions

Liver cancer remains a significant global health challenge with limited therapeutic options. A deeper understanding of the mechanisms underlying liver tumor initiation and progression can facilitate the development of personalized medicine strategies. Among experimental models, DEN-induced mouse liver cancer closely resembles human HCC of poor prognosis, both in global gene expression patterns and histological phenotypes (Kamino et al. 2011; Lee et al. 2004). Using cDNA microarray analysis, we demonstrated that HLJ1 deficiency alters hepatic gene signatures associated with chemical-induced liver cancer and IL-6/STAT3 signaling, and HLJ1 knockout mice showed pronounced STAT3 activation in normal liver adjacent to DEN-induced tumors. While STAT3 is recognized as a vital oncogene within liver tumors, its role in peritumoral regions has been less explored. Our results suggest that HLJ1-deficient livers provide a growth-advantageous premetastatic niche that promotes tumor progression. STAT3 in CD68^+^ myeloid cells has been identified as a potential marker for premetastatic niche detection and monitoring. The recruitment of these cells to premetastatic niches and future tumor outgrowth relies on activated SIPR1-STAT3 signaling (Deng et al. 2012; Zhang et al. 2013). STAT3 activation in hepatocytes establishes a pro-metastatic and growth-supportive niche in the liver (Lee et al. 2019). Moreover, STAT3 may promote tumor progression by modulating the immune response within tumor microenvironments through several signaling pathways, including S1PR1, JAK, and IL-6 (Wang et al. 2024; Zhong et al. 2024). These implicates that HLJ1 might suppress tumor metastasis and outgrowth by inhibiting STAT3 activation and preventing premetastatic myeloid cell colonization. Since we observed that HLJ1 expression was negatively correlated with SOCS3 but not with IL-6, LIF, ROS, or EGFR, further analysis of JAK1/2 activation could offer deeper insights into the molecular mechanisms by which HLJ1 regulates peritumoral STAT3 activation and subsequent liver cancer niche formation.

Recent studies have highlighted the important roles of HSPs in DEN-induced liver carcinogenesis (Rachidi et al. 2015; Sakurai et al. 2008), implicating HLJ1, a member of the HSP family, as a potentially indispensable factor in hepatocarcinogenesis. Of note, although HLJ1 can be upregulated in response to various environmental stresses, such as high-fat treatment or lipopolysaccharide exposure (Luo et al. 2022; Luo et al. 2018), its expression remained unchanged after short-term DEN administration (Fig. 2D). This suggests that its expression may be selectively regulated by specific stressors rather than by chemical carcinogens in the early stages of exposure. In a short-term DEN study, we found that HLJ1 mediated DEN-induced DNA damage since *Dnajb4*^–/–^ mice showed not only hepatic gene signatures related to DNA damage response but also up-regulated H2AX phosphorylation in the liver after DEN administration. Enrichment analysis of HLJ1-related genes in clinical tumor tissues suggests that HLJ1 may activate the intra-S checkpoint in response to DNA damage during the S phase, thereby protecting genomic integrity and ensuring replication fidelity. Additionally, HLJ1 may play a role in responding to double-strand breaks and regulating homologous recombination for DNA repair. A previous study has shown that HLJ1 is a caspase-3 substrate and its expression enhances UV-induced apoptosis in NSCLC (Lin et al. 2010). However, we found that HLJ1 deletion did not impact apoptosis, as indicated by similar levels of cleaved caspase-3, bax, and serum ALT in the short-term DEN study. Nevertheless, the proliferation marker was significantly upregulated in tumors following HLJ1 deletion, indicating that HLJ1 may suppress tumorigenesis by inhibiting DEN-induced compensatory proliferation rather than promoting tumor cell apoptosis.

Although the tumor-suppressive role of HLJ1 has been highlighted in several cancer types (Chen et al. 2023; Liu et al. 2014; Miao et al. 2018; Tsai et al. 2006), it may enhance tumor metastasis in HBV-induced liver cancers (Zhang et al. 2011). Importantly, these studies primarily focused on mature or late-stage tumor cells, whereas our study aimed to investigate HLJ1’s role in adjacent normal tissues, during carcinogenesis initiation, and potential cancer prevention. Regarding tumor regions, we found tumor size and number increased in HLJ1-deficient mice, consistent with its tumor-suppressive role. Although HLJ1 levels were relatively low in normal tissues (Fig. 4), it is possible that HLJ1 was induced in tumor tissues to counteract tumorigenesis-related stress. In normal tissues, lower p-STAT3 levels in *Dnajb4*^+*/*+^ mice compared to *Dnajb4*^*–/–*^ mice suggest that the absence of HLJ1 may enable sustained tumor progression. Further experiments are needed to verify these findings. In tumor tissues, pathways commonly involved in HCC carcinogenesis, such as ERK, AKT and STAT3, showed no differences following HLJ1 ablation (Fig. 5A). Previous studies have linked HLJ1 to additional pathways, including Src and the IL-12/IFN-γ axis (Chen et al. 2016; Luo et al. 2022). Future research should explore whether IL-12 plays a role in exacerbating hepatocarcinogenesis in HLJ1-deficient livers via immune modulation. Although the peritumoral expression of p-STAT3 was higher in *Dnajb4*^–/–^ mice, it shows minimal differences between the tumor sections of *Dnajb4*^–/–^ and wild-type mice, suggesting another possibility that HLJ1 in the tumor regions may regulate tumor proliferation in a STAT3-independent manner.

We validate the role of HLJ1 in the peritumoral microenvironment utilizing the lung cancer cell line and melanoma cell line in syngeneic C57BL6 mice. Both tumor cells were derived from C57BL/6 wild-type mice, so their implantation can bypass host immune surveillance. While both cell lines are widely used in allogeneic cancer implantation models, we acknowledge that an orthotopic injection using a liver cancer cell line would provide more direct support for our findings and should be considered in future studies. Despite the multiple experimental models employed, certain limitations must be acknowledged. Although hepatocytes play a central role in HCC development, we cannot rule out the possibility that HLJ1 deletion exacerbated DEN-induced carcinogenesis through mechanisms beyond hepatocytes. Potential contributions from Kupffer cells, fibroblasts, oval cells, or endocrine signals warrant investigation. Future studies utilizing tissue-specific knockout models could help clarify these possibilities.

**Fig. 7:**
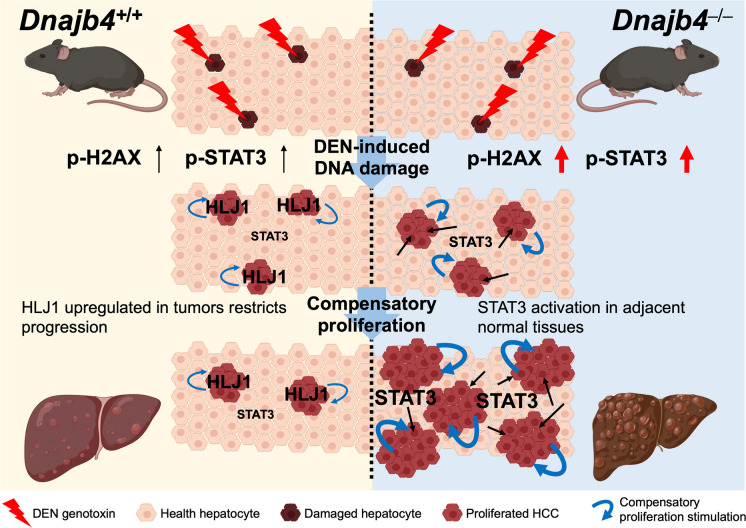
Schematic diagram illustrating enhanced hepatocarcinogenesis with abnormal STAT3 activation in adjacent normal tissues lacking HLJ1

Deletion of HLJ1 in peritumoral hepatocytes prevented tumor growth inhibition and resulted in a greater tumor load. A recent study demonstrated that peritumoral activation of the Hippo pathway effectors YAP and TAZ suppresses liver cancer in mice (Moya et al. 2019). YAP/TAZ can be recruited by STAT3/JUNB, mediating downstream cellular transformation associated with poor cancer survival (He et al. 2021). STAT3-YAP/TAZ signaling also plays a critical role in endothelial cells during tumor angiogenesis (Shen et al. 2021). Studies linking STAT3 to the Hippo pathway suggest that the peritumoral STAT3 activation could be associated with Hippo pathway activation, leading to HLJ1-mediated tumor progression. Given that HLJ1 may act as a tumor suppressor by dampening tumor growth through STAT3 activation inhibition, further research is essential to elucidate the complex crosstalk between peritumoral HLJ1/STAT3 signaling and liver tumors. In conclusion, we explored the crucial role of HLJ1 in alleviating DEN-induced carcinogenesis through multiple mechanisms: DNA damage attenuation, inhibition of compensatory proliferation, and down-regulation of phosphorylated STAT3 in normal liver tissue, ultimately reducing tumor burden (Fig. 7). Given that several strategies have been developed to induce HLJ1 expression, HLJ1 targeting represents a promising molecular approach for developing anticancer strategies for human liver cancers. Future clinical studies are imperative to further clarify HLJ1 expression and the underlying key pathways in both tumor and adjacent normal tissues from HCC patients, potentially opening new avenues for therapeutic intervention.

## Materials and methods

### Animal maintenance and treatments

HLJ1 knockout (*Dnajb4*^–/–^) mice were generated and maintained as previously described (Luo et al. 2022). Mice were housed in a pathogen-free facility with filter-topped cages, maintained under a standard 12-h light–dark cycle, and provided with standard rodent chow and water ad libitum. For the short-term experiment, 6–8-week-old mice received a single intraperitoneal injection of diethylnitrosamine (DEN, sigma #N0756) at 100 mg/kg. Liver tissues were collected and analyzed at 24 and 48 h post-administration (Chen et al. 2015; Naugler et al. 2007). For the long-term hepatocarcinogenesis experiment, male wildtype (*Dnajb4*^+/+^) and *Dnajb4*^–/–^ mice received an initial intraperitoneal injection of 20 mg/kg DEN at 15 days of age, followed by weekly 50 mg/kg DEN administrations between 4 to 11 weeks of age. Animals were sacrificed at 30 weeks of age (Memon et al. 2020). In the phenobarbital (PB) carcinogen experiment, mice received a single dose of DEN at 20 mg/kg at day 15, followed by 500 ppm phenobarbital in drinking water starting at day 28 until sacrifice at 52 weeks of age. All experimental procedures were approved by the Institutional Animal Care and Use Committee (IACUC), College of Medicine, National Taiwan University (IACUC number 20180126).

Immediately following sacrifice, whole liver were excised, captured, and weighed. At least three largest visible tumors and corresponding adjacent normal tissues were snap-frozen. The smallest lobe was reserved for RNA and protein extraction. For long-term DEN study, the externally visible tumors > 1 mm were counted using stereo microscopy. Tumor incidence rate, multiplicity, and volume were evaluated as (number of tumor-bearing mice) / (number of total mice), (tumor count) / (number of tumor-bearing mice), and (1/2*length*width^2), respectively. The largest lobes were fixed in 10% formalin for 24 h, paraffin-embedded and sectioned where 4-μm thick sections were H&E stained and examined for microscopic foci.

### cDNA microarray analysis

Gene expression microarray samples were obtained from 6–8-week-old wild-type and *Dnajb4*^–/–^ mouse livers. Total RNA was extracted from homogenized liver tissues using 1 mL TRI Reagent (Sigma, #T9424). For the reverse transcription, 2 ug RNA was converted to cDNA using High-Capacity cDNA Reverse Transcription Kits (Applied Biosystems, #4,368,814). Microarray analysis was performed using the Affymetrix Mouse Genome 430 2.0 Array. Raw gene expression data were processed and analyzed using GeneSpring Software. Data normalization was conducted using the GC Robust Multi-array Average (GCRMA) method with global median centering. The mean gene expression was calculated for genes with more than 1 probe set. Enrichment analysis was performed using GSEA software while pathway analysis was conducted through the MetaCore platform. The complete microarray dataset, including both raw and processed files, has been deposited in the GEO database under the accession number GSE148204.

### Immunoblotting experiments

Liver tissue (approximately 25 mg) was homogenized in a lysis buffer composed of 0.5 mL T-PER Tissue Protein Extraction Reagent (Thermo, #78,510), 1X PhosStop (Sigma, #04906845001) phosphatase inhibitor, 1X protease inhibitor cocktail (Sigma, #SI-s8830) and beads. Tissue disruption was performed using a TissueLyser II homogenizer (QIAGEN), with two cycles of homogenization at 30 Hz for 2 min, followed by a 15-min incubation on ice. Lysed samples were then centrifuged at 10,000 g for 5 min at 4˚C to remove tissue debris. The supernatants were collected and protein concentrations were determined using Pierce BCA Protein Assay Kit (Thermo, #23,225). 40 ug protein was mixed with 4 × sample buffer and 2-mercaptoethanol (Sigma, #M3148), then denatured by heating to 95˚C for 5 min. Protein separation was performed using 10% polyacrylamide gel (SDS-PAGE) at 100 V for 2.5 h. Proteins were transferred to 0.45 μm PVDF membranes (Millipore) at 35 V for 16 h. Immunoblotting was conducted using the following primary antibodies: anti-GAPDH, β-actin, HLJ1 (all from Proteintech); p-AKT, AKT, p-ERK, ERK, p-STAT3, STAT3, PCNA, and p-H2AX (all from Cell Signaling). Protein bands were semi-quantified using ImageJ Software.

### Immunohistochemistry staining

Tissue Sects. (4 μm thick) were deparaffinized and rehydrated through a graded series of solvents, followed by washing with double-distilled water. Antigen retrieval was performed by boiling the slides in sodium citrate buffer (pH 6.0) at 95–100 °C for 10 min, after which they were allowed to cool at room temperature for 20 min. The slides were washed twice with 0.05% TBS-Tween20 for 2 min. Endogenous peroxidase activity was blocked using 3% H_2_O_2_. Slides were incubated with diluted antibodies at 4˚C overnight, washed, and incubated with Histofine Simple stain Mouse MAX PO secondary antibody (Nichirei) for 30 min at RT. Chromogenic detection was performed by adding substrate for 5–20 min at RT, followed by counterstaining with hematoxylin for 1 min. Primary antibodies: HLJ1 (Proteintech), CK8/18 (Abcam), and glutamine synthetase (Abcam).

### Intrasplenic injection for tumorigenesis

LLC and B16F1 cell lines were cultured in DMEM (Thermo Fisher Scientific) supplemented with 10% FBS (Merck Millipore), 100 units/mL penicillin, 100 µg/mL streptomycin, 0.25 µg/mL Amphotericin B (Thermo Fisher Scientific), and 2 mM L-glutamine (Thermo Fisher Scientific) at 37 °C and 5% CO_2_. Before intrasplenic injection, cancer cells were suspended in HBSS. 6–8-week-old mice were anesthetized and practiced a 1 cm laparotomy and the spleen was exposed. The spleen was tightly ligated with a 6.0 silk suture at a 2–3 mm distance from the distal end. The needle was stuck into the ligated end and 3 × 10^5^ cells/mouse were transferred. Mice were sacrificed and the pathological change of livers was analyzed 13 and 18 days after B16F1 and LLC cell transplantation, respectively.

### Clinical dataset analysis

HLJ1 expression levels between tumor and normal or between HCC stages were compared using GEPIA where 369 tumor samples and corresponding 50 adjacent normal liver tissues from the TCGA database were analyzed (Tang et al. 2017). For enrichment analysis, top 1000 genes similar to HLJ1 from GEPIA HCC database were analyzed through the MetaCore platform.

### Statistical analysis

Statistical analysis was performed using the two-tailed, non-paired Student’s t-test, assuming equal variance including tumor volume and tumor size. HLJ1 expression levels between tumor and normal parts were compared using paired Student’s t-test. The correlation between HLJ1 and studied genes was evaluated using Pearson’s correlation coefficient. Differences were considered statistically significant when *P* ≤ 0.05.

## Supplementary information

Below is the link to the electronic supplementary material.

ESM 1 (DOCX 14.7 MB)

## Data Availability

The microarray data was deposited to GEO as raw and processed files with accession number GSE148204. Other data is provided within the manuscript or supplementary information files.
