# Supplementary material for: DNAJB4/HLJ1 deficiency sensitizes diethylnitrosamine-induced hepatocarcinogenesis with peritumoral STAT3 activation
Source: Cell Biol Toxicol. 2024 Dec 30;41(1):20. doi: 10.1007/s10565-024-09978-y (PMC11685265; doi:10.1007/s10565-024-09978-y)
Supplement: Supplementary file 1 — (DOCX 14.7 MB) [file 10565_2024_9978_MOESM1_ESM.docx]

**SUPPLEMENTAL MATERIALS**

**DNAJB4/HLJ1 deficiency sensitizes diethylnitrosamine-induced hepatocarcinogenesis with peritumoral STAT3 activation**

Wei-Jia Luo^1^, Wei-Lun Hsu^1^, Chih-Yun Lu^1^, Jung-Hsuan Chang^1^, Min-Hui Chien^1^, Kang-Yi Su^1,2^*

1. **Supplementary figure**

**1.1 Additional file 1: Supplementary Fig. S1**

**1.2 Additional file 2: Supplementary Fig. S2**

**1.3 Additional file 3: Supplementary Fig. S3**

**1.4 Additional file 4: Supplementary Fig. S4**

**1.5 Additional file 5: Supplementary Fig. S5**

**1.6 Additional file 6: Supplementary Fig. S6**

**1.7 Additional file 7: Supplementary Fig. S7**

**2. Supplementary table**

**2.1 Additional file 8: Supplementary Table S1**

**2.2 Additional file 9: Supplementary Table S2**

**Supplementary Figures**
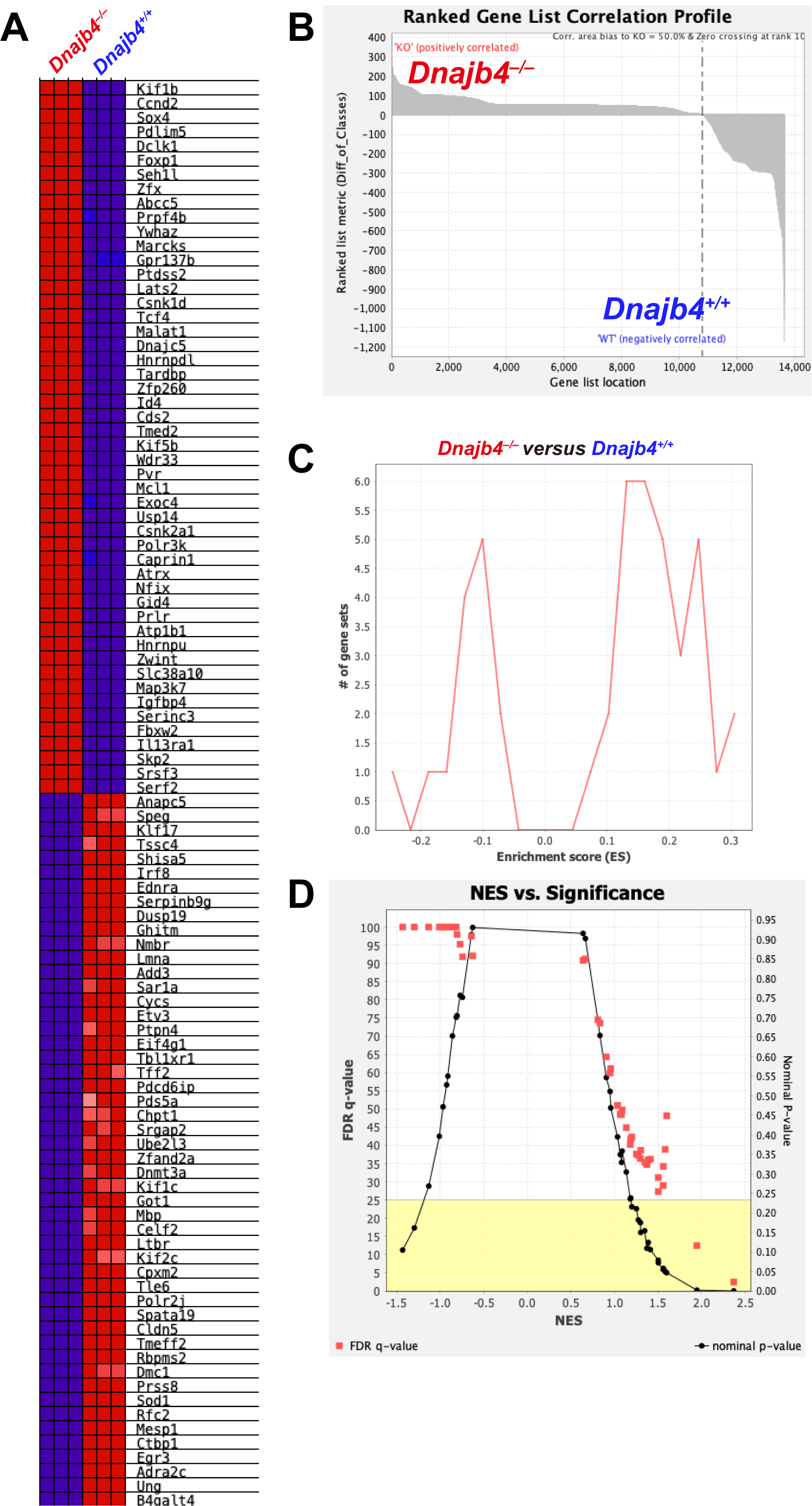


**Fig. S1. Gene set enrichment analysis discovered significantly enriched gene sets.** mRNA from the liver of *Dnajb4*^+/+^ and *Dnajb4*^–/–^ mice of age 6-8 weeks (n=3 per group) was extracted for cDNA microarray analysis and normalized intensity was used for GSEA analysis. (A) Heat map of the top 50 features for each phenotype. (B) Ranked gene list correlation profile. (C) Global enrichment histogram. (D) Plot of p-values vs. NES.


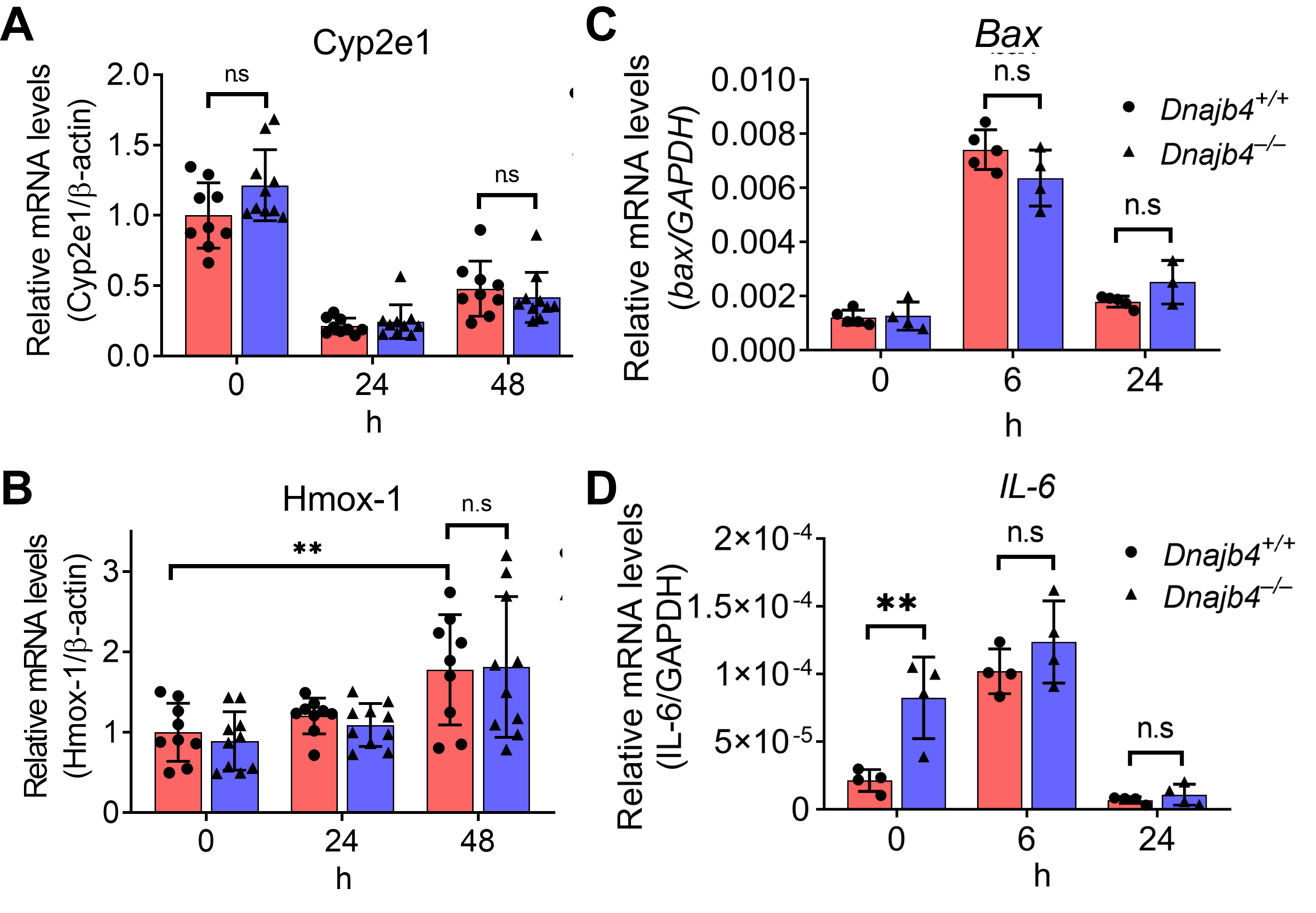


**Fig. S2. Cyp2E1, Hmox-1, Bax and IL-6 expression levels showed no difference between genotypes**. *Dnajb4*^+/+^ and *Dnajb4*^–/–^ mice were injected with a single dose of DEN (100 mg/kg) at age 6-8 weeks and sacrificed at indicated time points, and mRNA was extracted from whole liver lysate for further quantitative PCR analysis for (A) Cyp2e1, (B) Hmox-1, (C) Bax and (D) IL-6.


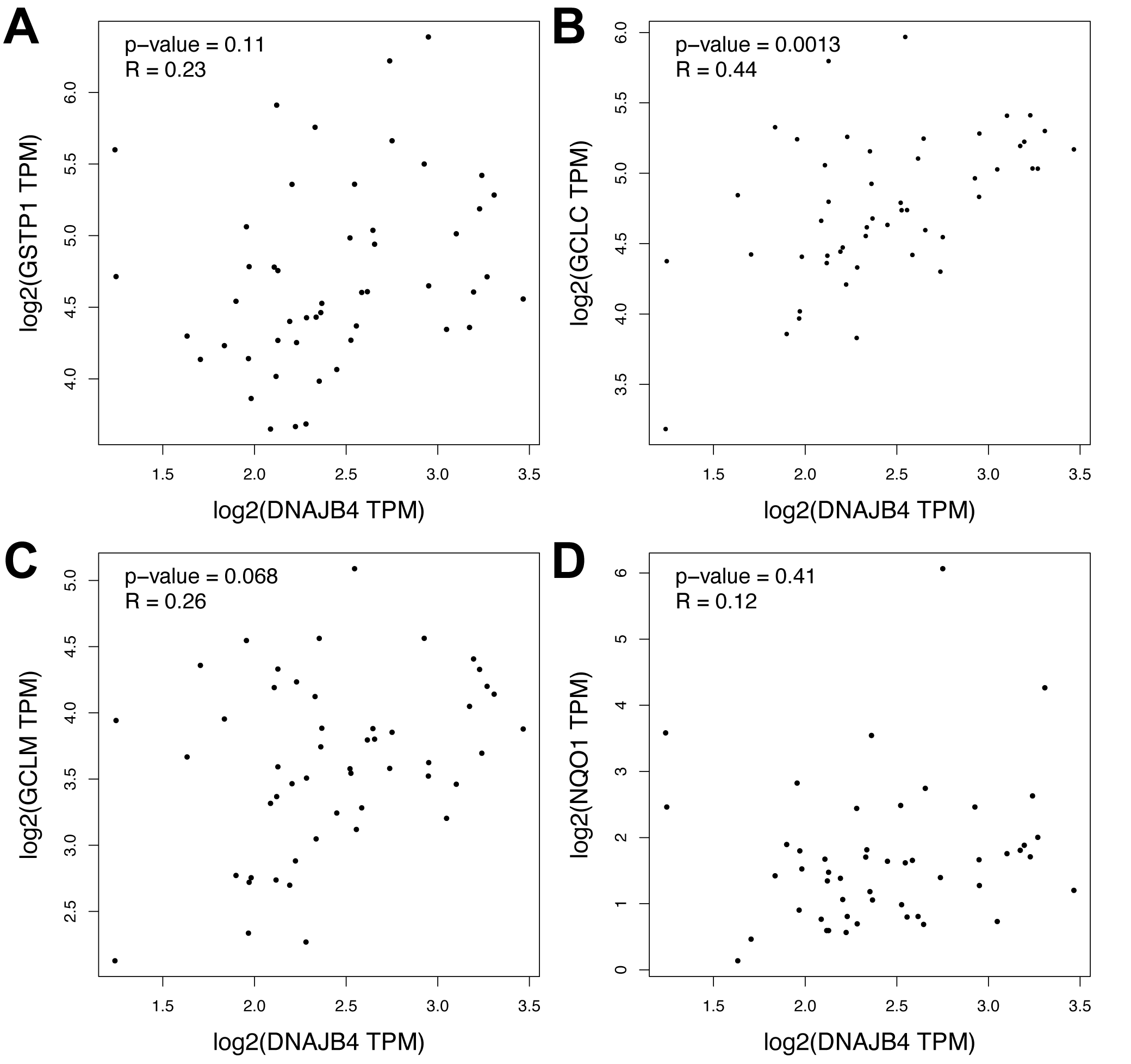
 **Fig. S3. Correlation analysis between oxidative stress markers and HLJ1.** 50 normal samples were analyzed from liver hepatocellular carcinoma TCGA database by using Pearson’s correlation analysis to measure the correlation between HLJ1/DNAJB4 and (A) GSTP, (B) GCLC, (C) GCLM, and (D) NQO1.


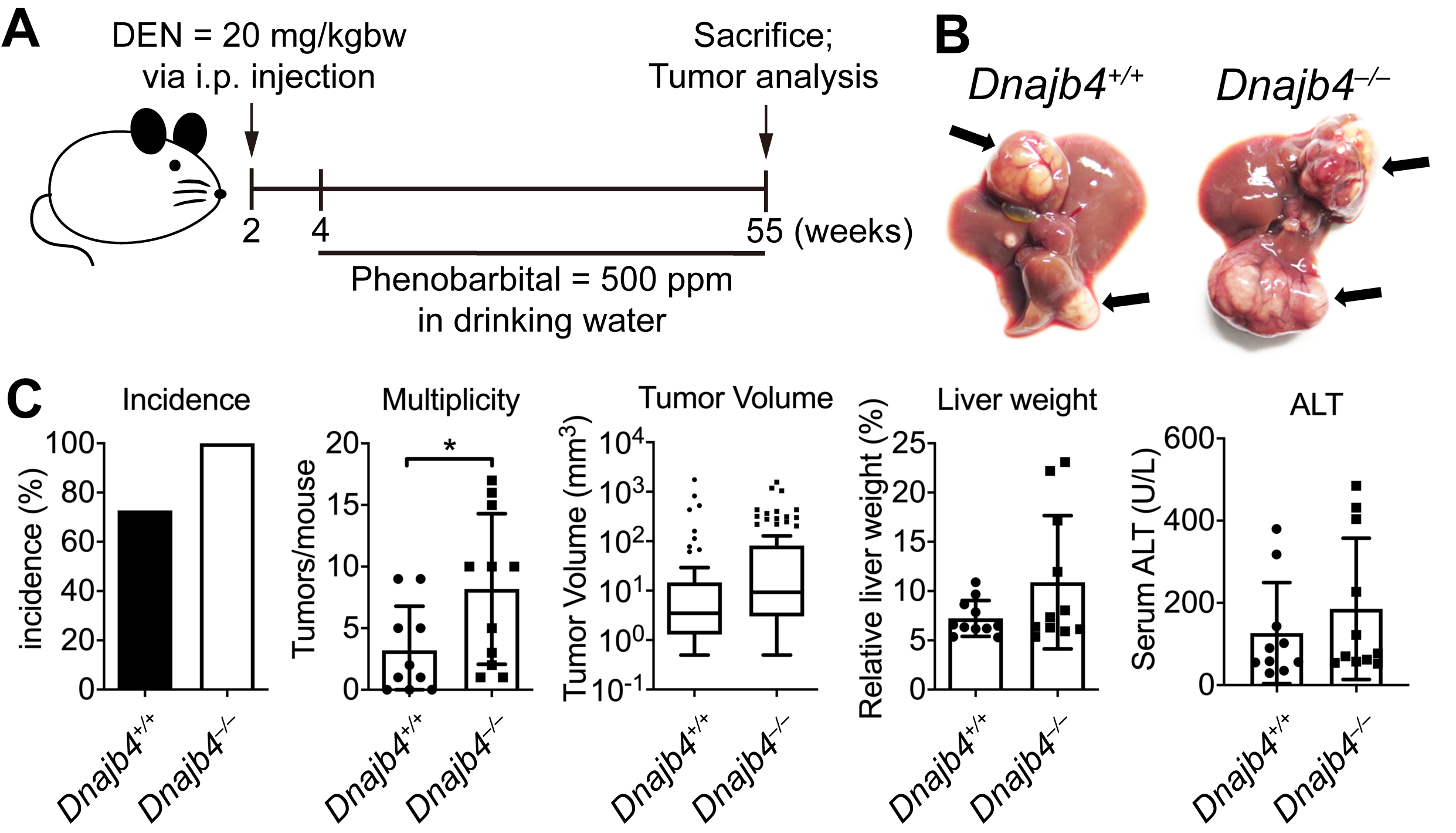


**Fig. S4. Increased HCC incidence and multiplicity in 55-week-old *Dnajb4*^–/–^ mice with DEN/PB treatment.** (A) *Dnajb4*^+/+^ and *Dnajb4*^–/–^ mice were injected 20 mg/kg DEN at age 2 weeks and fed with 500 ppm phenobarbital (PB) in drinking water ad libitum from age 4 until sacrifice at age 55 weeks for tumor evaluation. (B) Livers of the male *Dnajb4*^+/+^ and *Dnajb4*^–/–^ mice at age 55 weeks. Arrows point to single macroscopic tumors. (C) Serum ALT, AST levels, tumor multiplicity (tumors per mouse), tumor volume (1/2*length*width^2), the incidence rate of HCCs (>0.5 mm), and relative liver weight (liver/body weight) in n=10~11 male mice per group. Data are mean±SD. Tumor volumes are presented as Whiskers Tukey box plots. *p≤0.05.


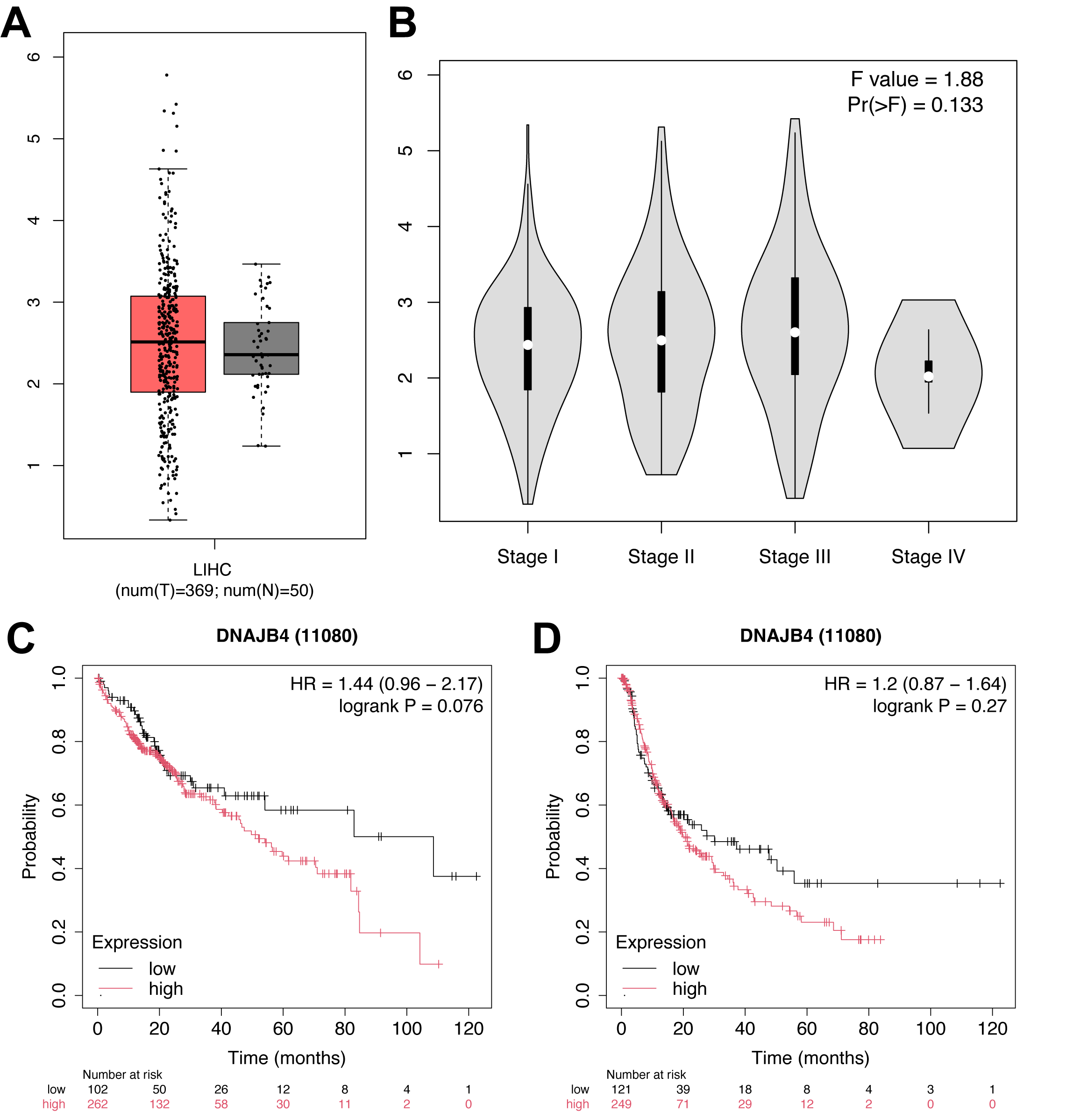


**Fig. S5. HLJ1 expression analysis from databases using clinical HCC specimens.** The TCGA database and GEPIA website were used to compare the difference in HLJ1 expression levels between (A) tumor and normal tissues or across (B) major HCC stages. The red box plot represented tumor tissues while the gray box plot represented normal tissues. Kaplan-Meier analysis was performed for the correlation between tumor DNAJB4/HLJ1 expression levels and (C) overall survival or (D) progression-free survival.


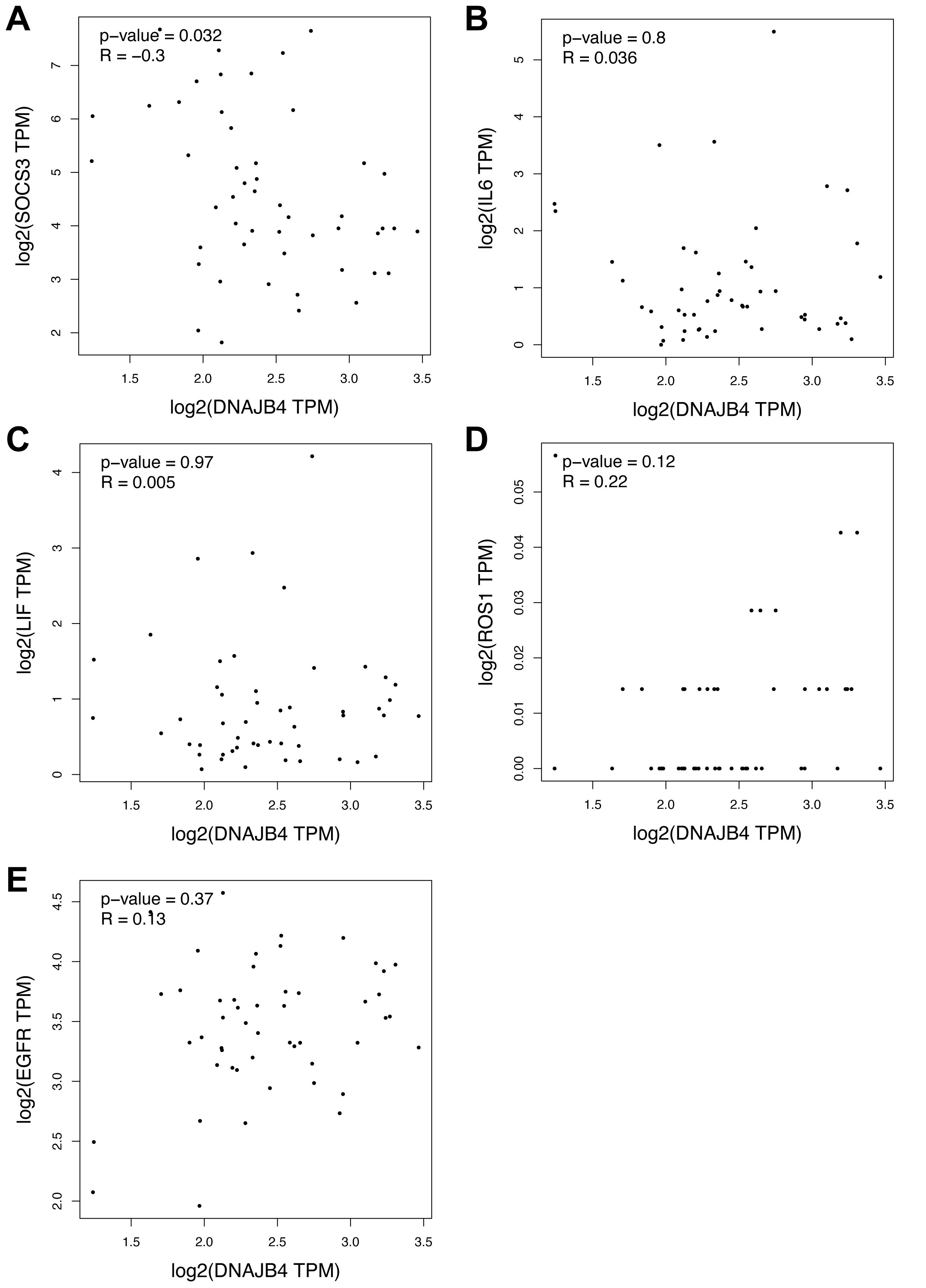


**Fig. S6. Correlation analysis between STAT3 signaling pathway and HLJ1.** 50 normal liver samples adjacent to tumors were analyzed from liver hepatocellular carcinoma TCGA database by using Pearson’s correlation analysis to measure the correlation between HLJ1/DNAJB4 and (A) SOCS3, (B) IL-6, (C) LIF, (D) ROS, and (E) NQO1.


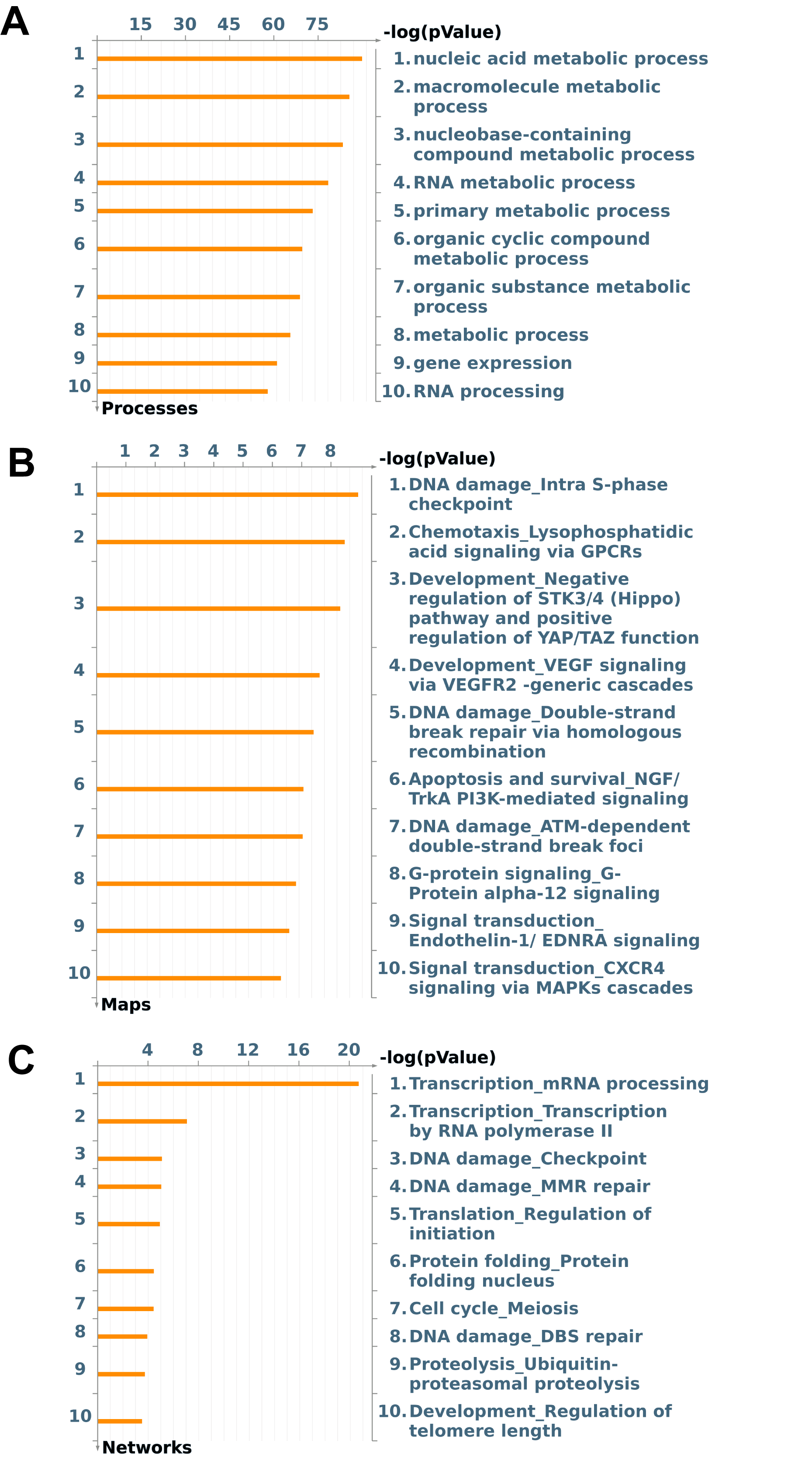


**Fig. S7. Genes similar to HLJ1 in tumor tissues were mainly enriched in pathways regarding metabolism and DNA damage.** Top 1000 genes most similar to HLJ1 in human tumor tissues were generated by the GEPIA and subsequently used for (A) gene ontology processes, (B) pathway maps, and (C) networks analysis by the Metacore

**Supplementary Tables**

| **Table S1.** Genes related to carcinoma incidence enrichment analysis between *Dnajb4*^+/+^ and *Dnajb4*^–/–^ by GSEA. | | | | |
| --- | --- | --- | --- | --- |
| SYMBOL | RANK IN GENE LIST | RANK METRIC SCORE | RUNNING ES | CORE ENRICHMENT |
| Nf2 | 66 | 206.1499 | 0.0201 | Yes |
| Trim37 | 89 | 201.8116 | 0.0435 | Yes |
| Stag1 | 107 | 199.1727 | 0.0673 | Yes |
| Sptbn1 | 129 | 194.1416 | 0.0907 | Yes |
| Smad4 | 191 | 177.6716 | 0.1113 | Yes |
| Prdx1 | 276 | 155.3635 | 0.1301 | Yes |
| Apc | 335 | 154.4469 | 0.1508 | Yes |
| Pten | 361 | 154.0929 | 0.1740 | Yes |
| Kras | 625 | 142.2922 | 0.1796 | Yes |
| Rev3l | 772 | 130.2915 | 0.1939 | Yes |
| Tes | 1216 | 103.1648 | 0.1863 | Yes |
| Cdkn1a | 1522 | 102.8732 | 0.1889 | Yes |
| Cbx7 | 1737 | 101.5061 | 0.1982 | Yes |
| Errfi1 | 1765 | 100.9773 | 0.2212 | Yes |
| Sav1 | 1893 | 98.5684 | 0.2368 | Yes |
| Trp73 | 1948 | 98.4500 | 0.2579 | Yes |
| Akt1 | 2068 | 98.2384 | 0.2741 | Yes |
| Tnk1 | 2218 | 96.1173 | 0.2882 | Yes |
| Stat3 | 2325 | 93.8741 | 0.3054 | Yes |
| Chuk | 2686 | 88.7323 | 0.3039 | No |
| Rb1 | 3274 | 62.4789 | 0.2857 | No |
| Tlr2 | 4581 | 51.6115 | 0.2147 | No |
| Braf | 5854 | 51.4750 | 0.1462 | No |
| Brca2 | 6157 | 51.4360 | 0.1490 | No |
| Mad1l1 | 6369 | 51.3975 | 0.1585 | No |
| Robo1 | 6478 | 51.3780 | 0.1755 | No |
| Cdkn2a | 6742 | 51.2598 | 0.1812 | No |
| Tff1 | 6758 | 51.2405 | 0.2051 | No |
| Htatip2 | 7830 | 46.8595 | 0.1513 | No |
| Pms2 | 8209 | 46.7034 | 0.1485 | No |
| Ssbp2 | 10182 | 11.4992 | 0.0285 | No |
| Fancd2 | 10450 | 4.6937 | 0.0339 | No |
| Pinx1 | 10864 | -5.0834 | 0.0285 | No |
| Apex1 | 10891 | -8.6320 | 0.0516 | No |
| Mus81 | 12383 | -258.7507 | -0.0330 | No |
| Hic1 | 12575 | -286.0206 | -0.0221 | No |
| Trp53 | 12784 | -290.4046 | -0.0124 | No |
| Mlh1 | 12985 | -292.5060 | -0.0021 | No |
| Pik3ca | 13128 | -296.8307 | 0.0125 | No |
| Men1 | 13556 | -590.8184 | 0.0061 | No |

| **Table S2.** Genes related to increased incidence of tumors by chemical induction enrichment analysis between *Dnajb4*^+/+^ and *Dnajb4*^–/–^ by GSEA. | | | | |
| --- | --- | --- | --- | --- |
| SYMBOL | RANK IN GENE LIST | RANK METRIC SCORE | RUNNING ES | CORE ENRICHMENT |
| Foxm1 | 250 | 162.1381 | -0.0099 | Yes |
| Ptpn11 | 279 | 155.0092 | -0.0033 | Yes |
| Apc | 335 | 154.4469 | 0.0012 | Yes |
| Pten | 361 | 154.0929 | 0.0080 | Yes |
| Runx1 | 412 | 151.3413 | 0.0129 | Yes |
| Ppp2r5a | 507 | 148.6966 | 0.0146 | Yes |
| Ppp2r1a | 749 | 131.5994 | 0.0054 | Yes |
| Lox | 860 | 121.9398 | 0.0059 | Yes |
| Flcn | 876 | 120.6671 | 0.0134 | Yes |
| Thbs2 | 1035 | 105.2937 | 0.0103 | Yes |
| Skil | 1169 | 103.2046 | 0.0091 | Yes |
| Mapk8 | 1219 | 103.1646 | 0.0141 | Yes |
| Bap1 | 1396 | 103.0089 | 0.0097 | Yes |
| Cdkn1a | 1522 | 102.8732 | 0.0091 | Yes |
| Tgfbr2 | 1605 | 102.6964 | 0.0117 | Yes |
| Trim16 | 1656 | 102.4414 | 0.0166 | Yes |
| Stk26 | 1661 | 102.4042 | 0.0249 | Yes |
| Ifnar2 | 1677 | 102.2286 | 0.0324 | Yes |
| Trp53inp1 | 1743 | 101.4661 | 0.0362 | Yes |
| Errfi1 | 1765 | 100.9773 | 0.0433 | Yes |
| Bcl2l14 | 1841 | 99.3398 | 0.0464 | Yes |
| Taf4 | 1844 | 99.2620 | 0.0549 | Yes |
| Tsc1 | 1845 | 99.2044 | 0.0635 | Yes |
| Gnai2 | 1865 | 98.7354 | 0.0707 | Yes |
| Ahr | 1872 | 98.6670 | 0.0789 | Yes |
| Hint1 | 1926 | 98.4905 | 0.0836 | Yes |
| Trp73 | 1948 | 98.4500 | 0.0906 | Yes |
| Bub3 | 2027 | 98.2968 | 0.0935 | Yes |
| Gpa33 | 2058 | 98.2566 | 0.0999 | Yes |
| Klf4 | 2169 | 97.3192 | 0.1004 | Yes |
| Cat | 2180 | 97.1428 | 0.1083 | Yes |
| Habp4 | 2226 | 95.9727 | 0.1136 | Yes |
| Ptprt | 2292 | 94.0511 | 0.1174 | Yes |
| Elavl1 | 2321 | 93.8775 | 0.1239 | Yes |
| Stat3 | 2325 | 93.8741 | 0.1323 | Yes |
| Bub1 | 2355 | 93.7774 | 0.1388 | Yes |
| Fgfr2 | 2483 | 93.1538 | 0.1380 | Yes |
| Yap1 | 2587 | 90.3066 | 0.1390 | Yes |
| Cuedc2 | 2830 | 84.2255 | 0.1298 | Yes |
| Pard3 | 2847 | 83.9868 | 0.1372 | Yes |
| Chfr | 2875 | 82.9111 | 0.1438 | Yes |
| Aldh2 | 3143 | 70.2888 | 0.1327 | Yes |
| Ttll3 | 3192 | 68.0859 | 0.1378 | Yes |
| Msh2 | 3310 | 60.8621 | 0.1377 | Yes |
| Cyp19a1 | 3530 | 55.6678 | 0.1302 | Yes |
| Rhob | 3541 | 55.0200 | 0.1380 | Yes |
| Slc7a11 | 3635 | 52.2400 | 0.1398 | Yes |
| Nfe2l2 | 3654 | 51.9232 | 0.1471 | Yes |
| Ranbp2 | 3709 | 51.8258 | 0.1517 | Yes |
| Poli | 3834 | 51.7480 | 0.1512 | Yes |
| Etv6 | 3927 | 51.7283 | 0.1530 | Yes |
| Ppard | 4288 | 51.6504 | 0.1350 | No |
| Wwox | 4291 | 51.6504 | 0.1435 | No |
| Cav1 | 4350 | 51.6316 | 0.1478 | No |
| Mif | 4733 | 51.5920 | 0.1282 | No |
| Nlrp6 | 5188 | 51.5338 | 0.1032 | No |
| Il9 | 5271 | 51.5337 | 0.1058 | No |
| Folr1 | 5395 | 51.5143 | 0.1053 | No |
| Klf10 | 5437 | 51.5143 | 0.1109 | No |
| Anp32b | 5469 | 51.5142 | 0.1172 | No |
| Cygb | 5541 | 51.5136 | 0.1206 | No |
| Pparg | 6010 | 51.4556 | 0.0946 | No |
| Ski | 6702 | 51.2984 | 0.0521 | No |
| Cdkn2a | 6742 | 51.2598 | 0.0579 | No |
| Ppp6c | 6750 | 51.2416 | 0.0660 | No |
| Trex2 | 6887 | 50.9861 | 0.0645 | No |
| Tnfsf10 | 6924 | 50.8896 | 0.0705 | No |
| Cebpa | 6960 | 50.7913 | 0.0765 | No |
| Chek2 | 7032 | 50.5762 | 0.0799 | No |
| Ncoa3 | 7035 | 50.5568 | 0.0884 | No |
| Map3k8 | 7189 | 49.4450 | 0.0857 | No |
| Nit1 | 7213 | 49.2249 | 0.0926 | No |
| Epha2 | 7219 | 49.1919 | 0.1008 | No |
| Stk11 | 7260 | 48.7728 | 0.1065 | No |
| Fos | 7315 | 48.0600 | 0.1111 | No |
| Chordc1 | 7342 | 47.7089 | 0.1178 | No |
| Tert | 7673 | 46.8987 | 0.1021 | No |
| Prkch | 7698 | 46.8984 | 0.1089 | No |
| Mbd3 | 7844 | 46.8593 | 0.1068 | No |
| Uimc1 | 7906 | 46.8207 | 0.1109 | No |
| Ltf | 8021 | 46.8006 | 0.1111 | No |
| Bub1b | 8066 | 46.7811 | 0.1165 | No |
| Fhit | 8160 | 46.7236 | 0.1182 | No |
| Pms2 | 8209 | 46.7034 | 0.1233 | No |
| Rassf1 | 8262 | 46.6640 | 0.1281 | No |
| Bin1 | 8330 | 46.4106 | 0.1317 | No |
| Ifnar1 | 8513 | 44.5876 | 0.1269 | No |
| Retnlb | 8672 | 42.3216 | 0.1238 | No |
| Akr1b8 | 8909 | 42.1268 | 0.1150 | No |
| Il12a | 9012 | 42.0490 | 0.1161 | No |
| Ercc2 | 9249 | 39.1645 | 0.1072 | No |
| Il22 | 9297 | 38.0791 | 0.1124 | No |
| Fpr2 | 9895 | 25.3699 | 0.0769 | No |
| Gast | 9990 | 20.6772 | 0.0785 | No |
| Cul9 | 10128 | 14.0812 | 0.0770 | No |
| Adamts18 | 10480 | 4.6743 | 0.0597 | No |
| Il22ra2 | 10546 | 4.6743 | 0.0635 | No |
| Xpa | 10908 | -10.8614 | 0.0454 | No |
| Cdkn1b | 10989 | -25.8884 | 0.0481 | No |
| Map3k6 | 11132 | -56.0438 | 0.0463 | No |
| Sf1 | 11233 | -89.2422 | 0.0475 | No |
| Egr1 | 11418 | -142.3297 | 0.0425 | No |
| Irf1 | 11461 | -151.7541 | 0.0480 | No |
| Tgfbi | 11513 | -168.0700 | 0.0529 | No |
| Rae1 | 11576 | -181.8955 | 0.0569 | No |
| Rnf20 | 11715 | -196.6263 | 0.0553 | No |
| Ceacam1 | 11863 | -227.9987 | 0.0531 | No |
| Mxi1 | 12157 | -243.2943 | 0.0400 | No |
| Usp24 | 12214 | -244.5517 | 0.0445 | No |
| Dek | 12504 | -281.2516 | 0.0318 | No |
| Ppargc1a | 12528 | -283.6737 | 0.0387 | No |
| Trp53 | 12784 | -290.4046 | 0.0284 | No |
| Vhl | 12829 | -290.8312 | 0.0338 | No |
| Stk38 | 12891 | -291.1818 | 0.0379 | No |
| Nmi | 13155 | -299.1516 | 0.0271 | No |
| Ptgs2 | 13518 | -578.1230 | 0.0089 | No |
